# Comparative Analysis of Frying Performance: Assessing Stability, Nutritional Value, and Safety of High-Oleic Rapeseed Oils

**DOI:** 10.3390/foods13172788

**Published:** 2024-09-01

**Authors:** Zhenglin Zhou, Pan Gao, Yuan Zhou, Xingye Wang, Jiaojiao Yin, Wu Zhong, Martin J. T. Reaney

**Affiliations:** 1 Key Laboratory of Edible Oil Quality and Safety for State Market Regulation, Key Laboratory for Deep Processing of Major Grain and Oil of Ministry of Education in China, College of Food Science and Engineering, Wuhan Polytechnic University, Wuhan 430023, China; 17786230050@163.com (Z.Z.); handsomejack-18@icloud.com (X.W.); yinjiaojiao@whpu.edu.cn (J.Y.); zhongwu@whpu.edu.cn (W.Z.); 2 Department of Food Science, University of Saskatchewan, Saskatoon, SK S7N 5A8, Canada; mjr997@mail.usask.ca; 3 Wuhan Institute for Food and Cosmetic Control, Wuhan 430012, China; zhouyuan860728@163.com; 4 School of Food Science and Technology, Jiangnan University, Wuxi 214122, China

**Keywords:** frying, high-oleic-acid rapeseed oil, harmful substances, antioxidant capacity

## Abstract

Frying is a critical process in the food industry, where selecting appropriate vegetable oils is key to achieving optimal results. In this study, French fries were fried at 175 °C with five different oils, the changes in the physicochemical indexes and free radical scavenging rate of the oils during the frying process were investigated, and the most suitable oils for frying were identified through comparative analysis using principal component analysis (PCA). We assessed the frying performances of hot-pressed high-oleic-acid rapeseed oil (HHRO), cold-pressed high-oleic-acid rapeseed oil (CHRO), soybean oil, rice bran oil, and palm oil utilizing principal component analysis over an 18 h period. The HHRO and CHRO showed lower acid values (0.31, 0.26 mg/g), peroxide values (2.09, 1.96 g/100 g), p-anisidine values (152.48, 178.88 g/mL), and total polar compound percentages (27.60%, 32.10%) than other oils. Furthermore, both the HHRO and CHRO demonstrated enhanced free radical scavenging abilities, indicative of their higher antioxidant capacities, as corroborated by the PCA results. Benzopyridine, 3-monochloropropane-1,2-diol ester, squalene, tocopherols, and polyphenol from the HHRO and CHRO during frying were compared. A comprehensive examination of harmful substances versus nutrient retention during frying revealed that the HHRO contained fewer hazardous compounds, while CHRO retained more nutrients. Therefore, this study analyzes the oxidation regulation of HHRO in frying applications, highlights the prospects of HHRO for frying in terms of health and economy, and contributes valuable insights for informed vegetable oil selection within the food industry.

## 1. Introduction

Frying is a vital culinary technique, which is valued for its simplicity and ability to enhance food flavors and odors [1]. As living standards rise and food preferences evolve, consumer focus on frying has increasingly shifted toward health, economy, and environmental impacts. The safety of fried foods, which is closely linked to consumer health, has recently garnered intense public attention [2]. Studies revealed that the type and quality of oils significantly affect the stability of fried foods, as oils undergo oxidation during frying [3]. During the frying process, oil will undergo a series of chemical reactions such as oxidation, hydrolysis, isomerization, and polymerization under the influence of high temperature, oxygen, moisture, and other factors. This can lead to color change, viscosity increase, quality degradation, and even the production of substances harmful to humans, including aldehydes, acids, ketones, alcohols, and others, such as trans fatty acids, triglyceride polymers, and acrylamide [3]. Saturated and unsaturated fatty acids affect the degradation of frying oils, and the presence of large amounts of polyunsaturated fatty acids positively correlates with oxidative degradation and affects the antioxidant properties between different fats and oils. To increase oxidative stability during frying, it is recommended to use oils with low levels of unsaturation [1,3]. Thus, understanding oil stability in frying is essential for selecting oils that improve fried food quality [4]. Additionally, the environmental impact of large-scale edible oil production and handling is crucial for selecting suitable frying oils.

Common frying oils like soybean oil (SO), palm oil (PO), and rice bran oil (RBO) are characterized by unique fatty acid profiles, stability, and nutrient contents. Their smoke points are higher than the temperatures typically used for frying, making them suitable for such applications [5,6]. High-oleic-acid rapeseed oil (HRO), with an oleic acid content of over 72%, is known for its exceptional oxidative stability [7]. Oleic acid, a monounsaturated fatty acid (MUFA), is more stable than other unsaturated fatty acids and has been associated with reduced blood cholesterol and cardiovascular protection [8,9]. HORO also contains low levels of erucic acid, which has been linked to the production of fatty deposits in the heart. Therefore, given its high-oleic-acid and low-erucic-acid contents, HORO is considered a healthy choice for consumers [10]. In recent years, the health effects of HRO have made it widely praised in Asia, especially in China, and has gradually become a research hotspot. In addition, high-oleic rapeseed has a high oil content and is usually mechanically pressed during the production of HRO, while solvent extraction is usually used to increase oil yield when processing other oilseed crops with fewer oils. This leads to a large amount of waste solvents in the oil production process, which pollutes the environment. The frying process requires a large amount of frying oil as a medium, and the lower environmental pollution of the preparation of HRO undoubtedly indicates its superior suitability for frying. With its increasing production and emerging role as a substitute for other fats, HRO’s suitability for frying, influenced by processing methods like cold and hot pressing, warrants comprehensive analysis [10,11,12]. The global consumption of frying oil is a significant aspect of the food industry, with deep-fried foods being a staple in many cultures. The choice of frying oil is crucial, as it directly impacts the cost-effectiveness of food production and the sustainability of agricultural practices. HRO is favored for its high smoke point and resistance to polymerization, which means it can be used for extended periods without the need for frequent replacement. This extends the oil’s lifespan, reducing waste and associated costs. Additionally, HRO’s lower levels of saturated fats and higher levels of monounsaturated fats contribute to a healthier food product, aligning with consumer trends toward healthier eating options. The environmental footprint of HRO is also a point of interest. Compared to conventional oils, HRO requires less land and water for cultivation, and it produces less waste due to its longer usage in frying processes. This sustainability aspect is supported by research that highlights the potential of HRO to reduce the environmental impact of food production (OECD-FAO Agricultural Outlook 2020–2029).

Frying oil quality is traditionally evaluated using indicators like acid value (AV), peroxide value (POV), p-anisidine value (p-AV), total polar compounds (TPC), and free radical scavenging rate, reflecting oil degradation and antioxidant capacity during frying [13,14]. Nutrient presence and hazardous substances are also critical quality parameters [15]. This paper conducts a comprehensive comparison of HRO against other edible oils based on these indices. We compared the fatty acid composition, frying stability, and antioxidant capacity of hot-pressed high-oleic-acid rapeseed oil (HHRO), cold-pressed high-oleic-acid rapeseed oil (CHRO), SO, RBO, and PO. Principal component analysis (PCA) was used to identify the two most promising oils. In addition, studies on minor-constituent compositions of high-oleic rapeseed oil during frying have been sparsely reported. By comparing the changes in antioxidant capacity, minor-constituent compositions and hazardous constituents during frying, we aimed to understand the oxidation regulation of high-oleic rapeseed oil during frying, which would help consumers better understand the frying performance of high-oleic rapeseed oil.

## 2. Materials and Methods

### 2.1. Samples and Reagents

Soybean oil (SO), rice bran oil (RBO), and palm oil (PO) samples were provided by Yihai Kerry Grain and Oil Co., Ltd. (Shanghai, China). High-oleic rapeseed (Jingmen, China), post-harvest, was immediately pressed using an electric screw press (J58-630T, Qingdao, China) at various temperatures. Oils with a pressing temperature below 60 °C are classified as cold pressed oils, while oils with a pressing temperature above 60 °C are classified as hot pressed oils. No heat treatment before cold pressing. Cold pressing was carried out at room temperature and the hot pressing temperature was set at 120 °C with a cake thickness of about 1–1.5 mm. Commercial precut potatoes (strips approximately 10 mm wide and 50 mm long) were purchased from a local supermarket (Wuhan, China). Chemical reagents and standards, including 2,4,6-tris(2-pyridyl) triazine (TPTZ), 2,2-diazo-bis-(3-ethylbenzothiazole-6-sulfonic acid), Trolox, 1,1-diphenylpicryl phenyl hydrazine (DPPH), 2,2-azinobis-3-ethylbenzothiazoline-6-sulfonate (ABTS), α-tocopherols standard (purity > 97%), γ-tocopherol standard (purity > 97%), 37-fatty acid methyl esters standard, hexane, KOH, and methanol were acquired from Sigma-Aldrich Co., Ltd. (Shanghai, China). Other necessary reagents, such as ethyl acetate, were analytically pure and were obtained from Sinopharm Chemical Reagent Co., Ltd. (Wuhan, China).

### 2.2. Frying Procedure

Frying experiments were conducted at 175 °C in a Frymaster fryer (Germany Pool, Hong Kong) with 2 L of oil adapted from Zou et al. [6]. French fries (5 × 1 × 1 cm) underwent a cycle of 1 min frying and 29 min waiting over 18 h. Over 18 h, 36 frying cycles were conducted, and the oil temperature was fixed at 175 °C. Oil samples (80 mL) were collected every 3 h, testing of oil samples every 6 frying cycles. No additional oil was added. Room temperature and humidity were not considered.

### 2.3. Fatty Acid

The fatty acid composition was determined by analysis of fatty acid methyl esters by gas chromatography using an Agilent 7890A (Agilent, Santa Clara, CA, USA) gas chromatography (GC) system with a flame ionization detector [6]. The oil samples (0.20 mg) were dissolved in 3 mL n-hexane and mixed with 0.4 mL 2 mol/L methanolic potassium hydroxide to prepare fatty acid methyl esters. The analytes were separated on a Trace TR-FAME capillary column (30 m × 0.25 mm, 0.25 μm, Agilent, Santa Clara, CA, USA). Nitrogen was used as the carrier gas with a flow rate of 1 mL/min and a split ratio of 100:1; the inlet temperature was 220 °C; the injection volume was 1 μL. The GC temperature program was as follows: the column was maintained initially at 100 °C for 13 min, increased to 180 °C with a rate of 10 °C/min, maintained at 180 °C for 10 min, then increased to 220 °C at 2 °C/min and held for 5 min at 220 °C. Fatty acids were identified by comparing retention times with 37 fatty acid methyl ester standards, and their proportions were quantified based on the percentages of peak area and response factors.

### 2.4. Basic Physiochemical Properties

The AV, POV, and p-AV were determined according to the Cd 3d-63, Cd 8b-90, and Cd 18–90, officially recommended by AOCS. The TPC content was estimated based on the dielectric constant using a deep-frying oil tester (Testo 270; Testo, Schwarzwald, Germany). The results of AV, POV, p-AV, and TPC were expressed as mg KOH/g, g/100 g, g/mL, and %, respectively.

### 2.5. Free Radical Scavenging Capacity

The ABTS, FRAP, and DPPH radical scavenging capacity tests were conducted according to Zhang et al. [14] with some modifications. Oil samples (1.0 g) and 4 mL methanol were pipetted into a 20 mL brown glass bottles, vortexed, and mixed with VORTEX-5 Vortex oscillator (Dam industry Co., Ltd., Shanghai, China) at 2000 r/min in the dark for 30 min. After settling the upper phase was taken as an extract of polar components. The above steps were repeated three times, with 5 mL methanol added each time and then the extracts and stored at 4 °C. The lower faction comprised a phase enriched in nonpolar substances. To prepare whole oil samples, oil samples (0.15 g) were dissolved in 10 mL of ethyl acetate. The ABTS solution and potassium persulfate solution were mixed in a brown volumetric flask and allowed to stand in the dark at room temperature for 12–16 h to prepare the stock solution. Before the determination, the reserve solution was diluted with methanol solution at 1:1 to obtain the ABTS free radical working solution.

#### 2.5.1. DPPH

Polar extract of oil samples (200 μL) and 6 mL chromatographic-grade methanol were added in a 10 mL brown glass tube. Then, 2.0 mL of the diluted polar phase and DPPH-methanol were taken and reacted in the dark for 2 h. The absorbance at 517 nm was determined (UV755B, Youke Instrumentation Co., Ltd., Shanghai, China). Prior to analysis of the nonpolar and whole oil extracts, samples were dissolved in ethyl acetate. Trolox was used as a standard and antioxidant activity was calculated and expressed as μmol TE/kg.

#### 2.5.2. ABTS

ABTS free radical working solution (2 mL) and 100 μL methanol extract of oil sample were combined in an 8 mL brown sample bottle and reacted for 20 min at 25 °C in the dark. The absorbance of the reaction solution was measured at 734 nm using a solution of methanol as a blank. Trolox was used as a standard and antioxidant activity was calculated and expressed as μmol TE/kg.

#### 2.5.3. FRAP

The antioxidant activity was evaluated using an FRAP (ferric-reducing antioxidant power) assay. The FRAP solution was prepared using a 0.1 mol/L sodium acetate buffer with a pH of 3.6. The absorbance of the FRAP solution was detected at a wavelength of 593 nm. Samples of the polar extract (200 μL) were diluted by adding 6 mL of methanol. Subsequently, 2.0 mL of this methanol dilution was mixed with the FRAP solution. The mixture was incubated at 37 °C for 30 min in a water bath. The absorbance of the resulting solution was measured using a UV755B spectrophotometer (Youke Instrumentation Co., Ltd., Shanghai, China). Trolox, a known antioxidant, was used as a standard for comparison. The antioxidant activity was then calculated and expressed in terms of Trolox equivalents per kilogram (μmol TE/kg).

### 2.6. Hazardous Substances

The concentration of benzopyrene (BaP) was determined using the method outlined in BS EN 16619:2015 [14]. The 3-monochloropropane-1,2-diol ester (3-MCPDE) test was conducted following the procedure described by Zhang et al. [14], with certain modifications. A Trace1310 ISQ gas chromatography analyzer (Thermo Fisher, Waltham, MA, USA) equipped with an HP-5MS capillary column (30.0 m × 0.25 mm × 0.25 μm, Thermo Fisher, Waltham, MA, USA) was used for the analysis of 3-MCPDE content. The column temperature was programmed to ramp up from 85 °C (held for 0.5 min) to 150 °C at a rate of 3 °C/min, then to 180 °C at a rate of 12 °C/min, and finally to 280 °C at a rate of 25 °C/min. The temperature was held at 280 °C for 5 min. The injection volume was set at 1 μL, with a split ratio of 20:1. Helium (99.99% purity) was used as the carrier gas, flowing at a rate of 1.0 mL/min.

### 2.7. Minor-Constituent

#### 2.7.1. Squalene

A sample of oil (0.20 g) was weighed and mixed with 50 mL of a 1 mol/L potassium hydroxide–ethanol solution. The mixture was saponified and subsequently extracted with hexane until the lower effluent became neutral. The hexane extract was then filtered and analyzed using an Agilent 6890N gas chromatograph (Agilent, Santa Clara, CA, USA) equipped with a flame ionization detector (FID). The compounds were separated on an HP-5 capillary column (0.25 µm, 30 m × 0.32 mm, Agilent, Santa Clara, CA, USA) under the following conditions: helium flow rate: 40 mL/min; split ratio: 1:10; initial column temperature: 160 °C; detector temperature: 300 °C; inlet temperature: 250 °C; and injection volume: 1.0 µL. 25 mg of squalane standard which was weighed into a 25 mL volumetric flask, and the solution was fixed with hexane to form a 1 mg/mL squalane internal standard solution. The mass of squalene in the sample was calculated based on the peak area ratio of squalene and squalane in the test solution, the mass of the internal standard, and the correction factor for squalene and squalane. The results were reported in mg/kg.

#### 2.7.2. Polyphenols

Polyphenols were analyzed using Folin–Ciocalteu’s reagent method [16]. A total of 1.0 g of oil was dissolved in 5 mL n-hexane and eluted over a solid-phase extraction column (Sepax, Newark, DE, USA) with 15 mL n-hexane. The polar oil extract residue was mixed with 0.5 mL of Folin–Ciocalteu reagent and 1 mL Na_2_CO_3_ (10%), and incubated in the dark for 2 h. The absorbance was measured at 765 nm, and the quantitative results were calculated using an analytical curve of gallic acid and expressed as mg of gallic acid equivalents per kg of oil sample (mg/kg).

#### 2.7.3. Tocopherol

The tocopherol content was determined following ISO 9936 [14], and analyzed by Agilent 1260 high-performance liquid chromatography (Agilent, Santa Clara, CA, USA) procedure with a G1321B fluorescence detector (Agilent, Santa Clara, CA, USA). The silica column (5 µm, 4.6 m × 250 mm, Hanbon, Huai’an, China) was used for chromatographic separation at 25 °C. The mobile phase was an n-hexane/deionized water (98/2, *v*) mixture at a flow rate of 1.2 mL/min. Peak quantification was performed at 285 nm. The standard of α-tocopherol and γ-tocopherol were quantified.

### 2.8. Statistical Analysis

Data were analyzed in triplicate with averages and their standard deviations computed using Microsoft Excel 2019 (Microsoft, Redmond, WA, USA). The data were characterized by one-way ANOVA analysis using Origin 2017 (Originlab, Northampton, MA, USA) and SPSS 19.0 (IBM, Armonk, NY, USA); Duncan’s multiple-range test was made a significant test of the different variables and significance test for differences among products. Principal component analysis (PCA) of the fatty acid composition, AV, POV, p-AV, TPC, and antioxidant capacity of the five oils was performed using SPSS 19.0, with inverse ratios used for negatively correlated factors. PCA is one of the most important methods for dimensionality reduction of variables. In practice, it is also common to perform principal component analysis on multiple variables to obtain more meaningful indicators, which transforms the original random vector whose components are correlated into a new random vector whose components are uncorrelated with the help of an orthogonal transformation. Through the statistical method of dimensionality reduction of PCA, multiple indicators were transformed into a few composite indicators, and the composite indicators were scored to select more suitable oils for frying.

## 3. Results and Discussion

### 3.1. Fatty Acid Composition

Table 1 illustrates the fatty acid composition of five oils, emphasizing oleic acid (C18:1, 27.26–78.93%), linoleic acid (C18:2, 6.60–47.98%), stearic acid (C18:0, 1.81–5.04%), and palmitic acid (C16:0, 3.28–39.01%) as key components. Notably, HHRO and CHRO displayed MUFA contents of over 75%, with C18:1 being predominant at 78.93% and 74.40%, respectively. This high level of C18:1, a known factor in reducing inflammation and cardiovascular risks [11], highlights the potential health benefits of HHRO and CHRO. Therefore, the high C18:1 and high MUFA contents of HHRO and CHRO reflected their nutritional value and safety. Furthermore, HHRO’s notably low polyunsaturated fatty acid (PUFA) content (6.73%) suggests a potential advantage in oxidative stability, which is crucial for maintaining oil integrity during high-temperature frying. The significant difference in SFA content between HHRO and CHRO may be attributed to the degradation of PUFA due to the instability of the unsaturated double bonds during high temperature processing, which resulted in a decrease in the proportion of PUFA and an increase in the proportion of SFA and MUFA, a hypothesis supported by García et al. [17]. Linoleic acid (C18:2), prone to oxidative degradation, led to secondary oxidation products and polar compounds, often used as indicators of fat deterioration [18]. HHRO, with the least amount of C18:2 (6.60%), suggested higher oxidative stability compared to the other oils, including SO with the highest C18:2 content (47.98%). The C18:2/C16:0 ratio, considered an indicator of PUFA deterioration [19], was significantly lower in HHRO (1.10) and PO (0.28) compared to the other oils.

### 3.2. Physicochemical Properties

**Figure 1:**
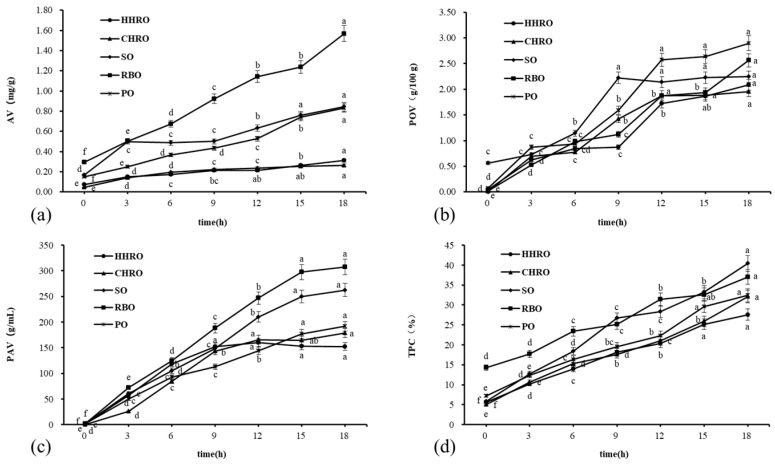
Changes in the five oils during the frying process: (**a**) changes in the acid values during the frying process; (**b**) changes in peroxide values during the frying process; (**c**) changes in p-anisidine values during the frying process; (**d**) changes in the total polar compounds during the frying process. The lettering of each point indicates that the differences between each group of samples at different times are significant (*p* < 0.05).

Figure 1 shows the physicochemical property analysis during frying. As depicted in Figure 1a, all oils maintained AV below the international standard limit (AV < 2.5 mg/g) set by Food Codex regulations post-frying. The AVs of CHRO (0.05–0.26 mg/g) and HHRO (0.07–0.31 mg/g) showed the slowest increments, with no significant difference between them. In contrast, RBO (0.30–1.57 mg/g) had the highest increase, diverging from the findings of Zou et al. [6], possibly due to differing frying durations. The AV content of other oils increased rapidly after frying times of more than 18h. The slow AV increase in the two HROs might be attributed to their low PUFA content, the oxidation rate of fatty acids increases with the increase in unsaturation, and HROs with the low PUFA content were less susceptible to hydrolysis and oxidation during frying [20]. Therefore, HRO demonstrated better frying stability over a short period of continuous frying.

Figure 1b shows the changes in POV during the frying process, which indicates the degree of oxidation of the oil during frying at high temperatures. After 18 h of continuous frying, the POV values of oils ranged from 1.96 (CHRO) to 2.90 (PO) mg/100 g. In addition, the increases in HHRO and CHRO were 2.04 mg/100 g and 1.94 mg/100 g, respectively, which were significantly smaller than those of the other three oil groups. This suggests that HHRO and CHRO possessed superior oxidative stability during the frying process. POV indicated the production of hydroperoxides, and unstable hydroperoxides were highly susceptible to decomposition into small molecules such as ketones, aldehydes, and acids at high temperatures [4]. Therefore, the growth of POV showed a fluctuating upward trend.

Figure 1c shows the p-AV value, which indicates the content of unsaturated aldehydes in the oil produced during secondary oxidation reactions. The p-AV was sorted by decreasing order from RBO (307.43 g/mL), SO (262.43 g/mL), PO (191.62 g/mL), CHRO (178.88 g/mL) to HHRO (152.48 g/mL) after frying 18 h. This indicates that HHRO and CHRO had fewer secondary oxidation products than the other three oils. The p-AV values increased rapidly during the initial 0-9 h of frying for HHRO and CHRO, followed by a more stabilized trend, attributed to the oxidation of fatty acids and the accelerated breakdown of hydroperoxides at elevated temperatures. Additionally, the high temperature likely caused the volatilization and decomposition of aldehydes [21], thereby affecting the p-AV trend.

As shown in Figure 1d, the TPC content all showed an increasing trend. TPC contains all of the generated polarity compounds such as monoglycerides, diglycerides, oxidized triacylglycerols, and triglyceride polymers. TPC, therefore, was considered one of the most objective indicators in evaluating the deterioration of deep-frying oils. The TPC content of the five oils increased to different degrees as the frying time increased. The international standard limits of the TPCs were 24–27% [22]; the TPC contents of SO, RBO, and PO reached its limit at 15 h; and HHRO and CHRO reached their limit for TPC content at 18 h. This shows that compared with the other three oils, HROs were discarded later after the frying process, which could control the production costs and have better frying applicability.

It is evident from the above changes in physicochemical properties that the two HROs outperformed the other three oils. The AV, POV, p-AV, and TPC of the five oils showed increasing trends during prolonged frying, which indicates the deterioration of the oils. Compared to the other three oils, HHRO and CHRO showed less deterioration, which indicates the better suitability and safety of HRO during prolonged frying.

### 3.3. Antioxidant Capacity Comparison

**Figure 2:**
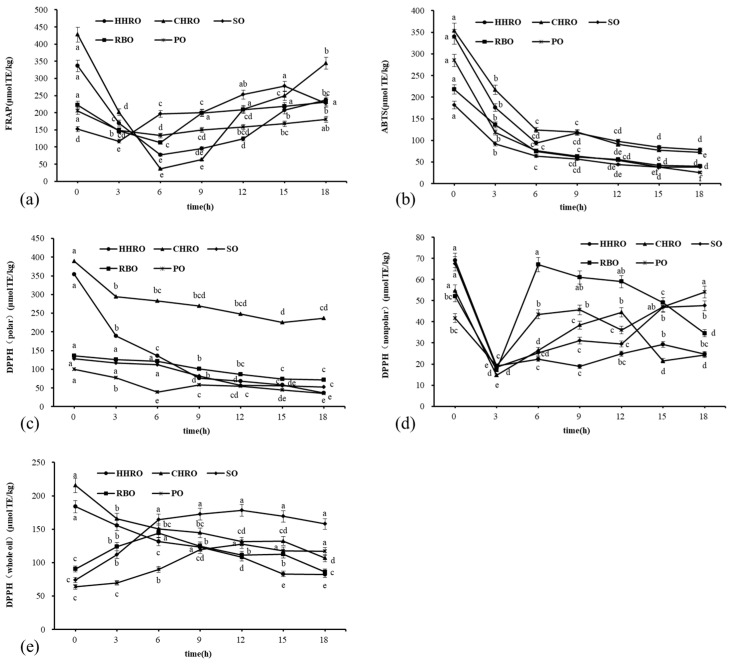
Changes in free radical scavenging during frying in five oils: (**a**) FRAP free radical scavenging capacity; (**b**) ABTS free radical scavenging capacity; (**c**) DPPH (polarity) free radical scavenging capacity; (**d**) DPPH (nonpolar) free radical scavenging capacity; (**e**) DPPH (whole oil) free radical scavenging capacity. The lettering of each point indicates that the differences between each group of samples at different times is significant (*p* < 0.05).

Free radical was generated during lipid oxidation, and their high reactivity with oxygen leads to their conversion to peroxides or hydroperoxides. These oxides are very unstable and can break down to produce more free radicals, which can trigger a chain reaction. Therefore, free radical scavenging ability is very important [23]. FRAP denotes the reduction capacity to reduce the TPTZ-Fe^3+^ complex to TPTZ-Fe^2+^ [24]. The data in Figure 2a show the FRAP free radical scavenging capacity of the oil samples, and the values of the five oils showed a fluctuating trend of decreasing and then increasing with prolonged frying. After 6 h of frying, the FRAP free radical scavenging capacity of the five oils increased slowly with the frying time. It might be due to a series of oxidation products generated during the frying process was also involved in the reduction of TPTZ-Fe^3+^ to TPTZ-Fe^2+^, which resulted in an increasing trend in the FRAP free radical scavenging capacity over time [25].

Different free radical scavenging methods denoted different antioxidant mechanisms, and the ABTS reaction was the reaction of the antioxidant with the ABTS free radical stock solution. As shown in Figure 2b, the ABTS free radical scavenging capacity of different oil samples showed a decreasing trend with prolonged frying. ABTS free radical scavenging capacity may be affected by antioxidant compounds such as phenols, tocopherols, and phytosterols, and depletion of antioxidant compounds during frying contributes to the decrease in ABTS [26]. What stands out is that the ABTS and FRAP of the two HROs were greater than those of the remaining three oils, both before and after frying, indicating that the two HROs had better free radical scavenging ability than the other three oils in the two free radical scavenging experiments described above.

The DPPH free radical scavenging capacity expresses the ability to reduce DPPH- to DPPH-H. As shown in Figure 2c, the DPPH radical scavenging capacity (polarity) of the HHRO and CHRO showed a rapid and then slow decrease with the prolonged frying time, and the other three oils showed a slow decrease with prolonged frying time. This might be attributed to the oxidization and decomposition of the antioxidant compound in HROs within the first 3 h [26]. What stands out is that the DPPH (polar) of the CHRO was significantly higher than the other four oils. This indicates that the CHRO had a stronger antioxidant capacity than the other four oils in terms of the DPPH (polar).

As shown in Figure 2d, the nonpolar DPPH radical scavenging capacity of all five oils showed a decreasing and then fluctuating increasing trend with the prolonged frying time. It is hypothesized that most of the minor-constituent compositions were soluble in the polar portion of the oil, and the nonpolar portion of the oil sample contained less minor-constituent compositions, the oxidation products generated during the frying process affected its nonpolar DPPH radical scavenging capacity [27]. After 18 h of frying, the DPPHs (nonpolar) of the HHRO and CHRO were not significantly different and lower than the other three oils. This indicates that the antioxidant capacities of the two HROs were lower than for the other three oils in terms of the DPPH (nonpolar).

Figure 2e shows the free radical scavenging capacity of DPPH (whole oil). The DPPH (whole oil) radical scavenging capacity of the two HROs showed a slight decrease with an increasing frying time. In contrast, the DPPH (whole oil) radical scavenging capacity of the other three oils showed an increasing and then decreasing trend with prolonged frying time. The decrease in the HHRO of the DPPH (whole oil) radical scavenging capacity from 0–3 h suggests that the antioxidant capacity of HRO was otherwise affected.

### 3.4. Principal Component Analysis

**Figure 3:**
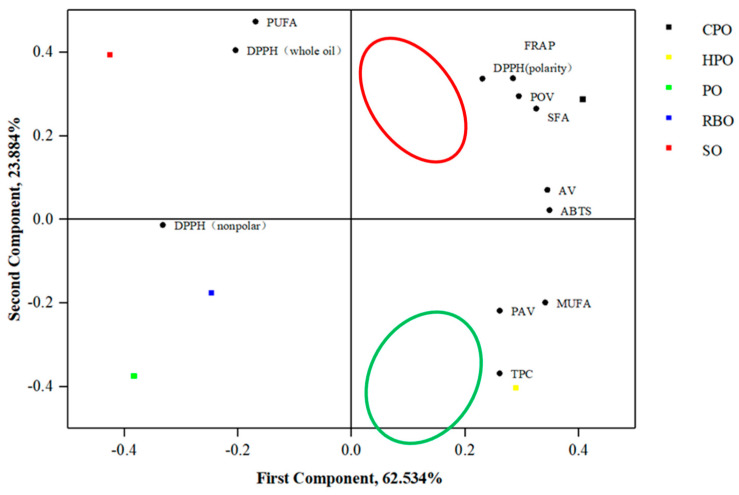
PCA loading plot of the five oils and projection of the variables onto the factor plot.

As shown in Figure 3, PCA was performed to assess associations among 12 quality indices for the five types of oil. Identified principal components (PCs) were considered to reflect all factors when the contribution of the total variance exceeded 85%. The first two components accounted for 100% of the total variability and were chosen appropriately. The first PC (PC1) contributed to 62.534% of the variance, reflecting the fatty acid composition and physicochemical properties. Among them, the physicochemical properties including AV, POV, p-AV, and TPC were of great significance in the oil stability and played a vital role in frying suitability. The second PC (PC2) contributed to 23.884% of the total change, reflecting the free radical scavenging capacity. Among them, free radical scavenging capacity including the FRAP, ABTS, DPPH (polarity), DPPH (nonpolar), and DPPH (whole oil) reflected the antioxidant capacity of the oil during frying. By standardizing the initial factor component matrix, the equation for each PC and comprehensive score formula could be obtained as follows:

F_1_ = 0.33X_1_ + 0.34X_2_ − 0.17X_3_ + 0.35X_4_ + 0.29X_5_ + 0.26X_6_ + 0.26X_7_ + 0.35X_8_ + 0.28X_9_ + 0.23X_10_ − 0.33X_11_ − 0.20X_12_

F_2_ = 0.27X_1_ − 0.20X_2_ + 0.47X_3_ + 0.07X_4_ + 0.29X_5_ − 0.22X_6_ − 0.37X_7_ + 0.02X_8_ + 0.34X_9_ + 0.34X_10_ − 0.01X_11_ + 0.40X_12_

Based on the above formula, the comprehensive score formula was as follows:

F = 0.7273F_1_ + 0.2727F_2_ = 0.31X_1_ + 0.19X_2_ + 0.01X_3_ + 0.27X_4_ + 0.29X_5_ + 0.13X_6_ + 0.09X_7_ + 0.26X_8_ + 0.30X_9_ + 0.26X_10_ − 0.25X_11_ − 0.04X_12_

The loading values of the PC were more closely associated with SFA, DPPH (polarity), FRAP, POV, and DPPH (nonpolar). Positive correlations were detected for SFA, DPPH (polarity), FRAP, and POV, whereas negative correlations were identified for DPPH (nonpolar). From the first principal component, it can be seen that the physicochemical properties, MUFA, SFA, and free radical scavenging ability (polarity) of the frying oils were positively correlated. This indicates that the oxidation level, polar antioxidant capacity, and SFA of frying oil were very important for the quality of the oil. Moreover, the total scores comprehensively, in descending order, were CHRO (2.857), HHRO (1.412), RBO (−1.040), SO (−1.252), and PO (−1.978). The high HHRO and CHRO scores are attributed to the low oxidation levels, high MUFA content, and polar antioxidant capacity. These characterization indicators reflect consumers’ quest for healthfulness and safety in frying oils. Consequently, the CHRO and HHRO were initially identified as optimal frying oils.

### 3.5. Harmful Substances

**Figure 4:**
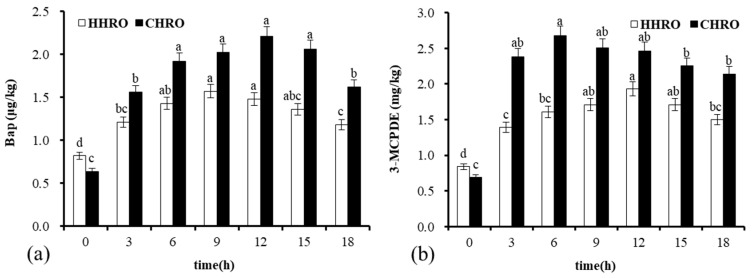
Contents of harmful substances during frying: (**a**) changes in benzopyridine during frying; (**b**) changes in 3-monochloropropane-1,2-diol ester during frying. Different letters indicate that the difference between each group of samples at different times is significant (*p* < 0.05).

The contents of harmful substances and the nutrient composition of frying oil are also a measure of the oil’s goodness. To further analyze the quality of HHRO and CHRO, changes in nutrients and hazardous substances during the frying process were examined. Figure 4a depicts the BaP of both oils following an increasing and then decreasing trend with prolonged frying time. According to EU NO835/2011 [14], the BaP content of edible oil should not exceed 2 μg/kg. The highest content of BaP in HHRO during frying was 1.57 μg/kg, which was below the limit. The BaP content of CHRO during frying for 9–15 h was beyond the limit of 2 μg/kg. This suggests that the differences in processing affected the BaP contents in HRO and HHRO, which had fewer harmful substances than CHRO during frying; this might be due to the high temperatures during hot pressing leading to the volatilization of BaP [11]. Moreover, the frying time for CHRO was recommended to not exceed 9 h to avoid surpassing the BaP limit.

3-MCPDE is a hazardous substance formed during food processing. As shown in Figure 4b, the 3-MCPDE content of two oils also showed an increasing and then decreasing trend with extended frying time, which might be due to the instability of 3-MCPDE at high temperatures, prolonged frying at high temperatures led to the oxidative decomposition of 3-MCPDE generated during the frying. The rate of decomposition of 3-MCPDEs exceeded the rate of their production, leading to a decrease in the content of 3-MCPDEs [28]. According to the EU 2020/1322 report [14], the 3-MCPDE content of edible oil should not exceed 2.5 mg/kg and the levels of 3-MCPDE in both oils were below the limit after frying. Moreover, the 3-MCPDE content of HHRO was consistently lower than that of CHRO after 3h of frying, which suggests that the hazardous substances in HHRO were lower than those in CHRO during the frying process. Long-term consumption of oils with high levels of BaP and 3-MCPDE carries the risk of carcinogenesis [28]. Therefore, choosing the right frying oil should not only take into account whether the amount of hazardous substances produced during frying exceeds the limit but also strictly control the frying time.

### 3.6. Minor-Constituent Compositions

**Figure 5:**
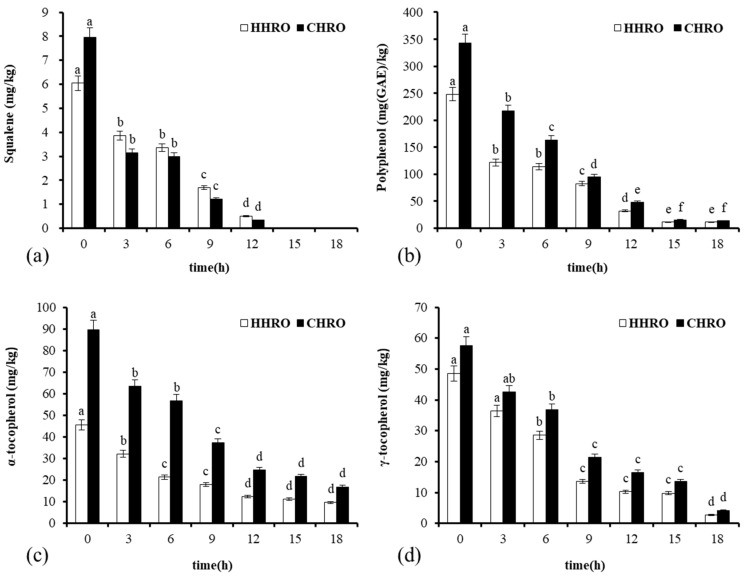
Contents of minor-constituent compositions during frying: (**a**) changes in squalene during frying; (**b**) changes in polyphenol during frying; (**c**) changes in α-tocopherol during frying; (**d**) changes in γ-tocopherol during frying. Different letters indicate that the difference between each group of samples at different times is significant (*p* < 0.05).

Squalene is known for its antioxidant properties [16]. As shown in Figure 5a, the squalene content of the two HROs showed a decreasing trend with an extended frying time, and the squalene content of CHRO decreased at a faster rate compared to HHRO. This aligned with the earlier observation regarding TPC, where the rapid increase in TPC in CHRO after 15 h may be attributed to the depletion of minor-constituent compositions in CHRO compared to HHRO. The squalene in both HROs became undetectable after 12 h of frying, which might be due to the poor thermal stability of squalene and easy to decompose into aldehyde and ketones under high temperatures [29]. Moreover, the initial values of squalene in CHRO were significantly greater than those in HHRO, which might be due to the high-temperature breakdown of the squalene in the oil during the hot-pressing process [30].

The variation in polyphenol contents during frying is shown in Figure 5b. The polyphenol content of the two oils decreased with the frying time, and there was no significant difference in polyphenol contents between the two oils after frying. What stands out is that the initial values of polyphenols in the CHRO were significantly greater than in the HHRO, which might be due to the loss of polyphenols at high temperatures [31]. During the pre-frying period, the HROs had higher levels of minor-constituent compositions. Antagonistic interactions between these compositions and antioxidant compounds in HROs could inhibit the bioactivity of antioxidants such as tocopherols [32], contributing to the reduced scavenging capacity of DPPH (whole oil) free radicals during the initial 0–3 h of frying.

The variation in tocopherol content during frying is shown in Figure 5c,d. The CHRO had high tocopherol content during frying for 0–3 h, enhancing its free radical scavenging capacity during the frying process [7]. This could explain the higher DPPH radical scavenging capacity (polarity) of the CHRO than those of the other four oils. The α-tocopherol and γ-tocopherol contents of CHRO were consistently higher than those of the HHRO during frying, which implies that CHRO has higher nutritional value. As shown in Figure 5d, the α-tocopherol depletion rates for the HHRO and CHRO were respectively 78.9% and 81.1%. As can be seen in Figure 5d, the γ-tocopherol depletion rates for the HHRO and CHRO were, respectively, 94.4% and 92.9%. It can be seen that the depletion rate of γ-tocopherol was significantly higher than that of α-tocopherol for both oils. This implies a higher antioxidant capacity of γ-tocopherol compared to that of α-tocopherol, and the more oxidizing γ-tocopherol participated in the oxidation reaction to be consumed first during the frying process [5]. The analysis of nutrients and hazardous substances revealed that the CHRO retained a higher level of nutrients, whereas the HHRO generated fewer hazardous substances during the frying process.

## 4. Conclusions

Our comprehensive analysis of five common frying oils identified HHRO and CHRO as superior options for frying, based on oil stability indexes through principal component analysis. The study further assessed these two oils for nutrient content and hazardous substances. By analyzing the physicochemical properties, antioxidant capacity, and nutrient and hazardous components of HRO during frying, we determined the good frying suitability of HRO. A higher antioxidant capacity and rich in minor-constituent compositions make HRO the preferred choice for consumers. The low contamination during processing and the simplicity of the extraction process make HRO advantageous for industrial mass production. However, this research has limitations. It focused solely on five oil samples, providing an incomplete result of the optimal oil for frying. The potential cost of the five frying oils from production and processing to the end of frying was not considered. Additionally, not all relevant frying metrics were considered, and in the future, it will be possible to delve deeper into more nutrients such as phytosterols and investigate the complex interactions between free radical scavenging rates and secondary ingredient composition. The multi-element study allowed us to refine the experiments. Future studies can also delve into the effects of different frying ingredients on HRO, enhancing consumer understanding of the health dimensions of HRO and expanding the market prospects for HRO.

## Figures and Tables

**Table 1:** Fatty acid composition (%) of five oils.

Fatty Acid	HHRO	CHRO	SO	RBO	PO
C16:0	6.01 ± 0.04 ^c^	3.95 ± 0.03 ^d^	11.83 ± 0.03 ^b^	3.28 ± 0.02 ^e^	39.01 ± 0.28 ^a^
C18:0	2.04 ± 0.01 ^d^	1.81 ± 0.01 ^e^	5.04 ± 0.08 ^a^	2.17 ± 0.02 ^c^	4.47 ± 0.03 ^b^
C18:1	78.93 ± 0.56 ^a^	74.40 ± 0.53 ^b^	27.26 ± 0.07 ^d^	44.48 ± 0.31 ^c^	44.48 ± 0.31 ^c^
C18:2	6.60 ± 0.05 ^d^	14.39 ± 0.10 ^b^	47.98 ± 0.07 ^a^	10.92 ± 0.08 ^c^	10.98 ± 0.08 ^c^
C18:3	0.13 ± 0.00 ^e^	3.04 ± 0.02 ^b^	6.09 ± 0.01 ^a^	2.27 ± 0.02 ^c^	0.17 ± 0.00 ^d^
C20:0	1.01 ± 0.01 ^a^	0.56 ± 0.00 ^c^	0.58 ± 0.01 ^bc^	0.59 ± 0.00 ^b^	0.39 ± 0.00 ^d^
C20:1	2.14 ± 0.02 ^b^	1.32 ± 0.01 ^c^	0.27 ± 0.01 ^d^	5.57 ± 0.04 ^a^	0.21 ± 0.00 ^e^
C22:0	2.35 ± 0.02 ^a^	0.33 ± 0.00 ^c^	0.70 ± 0.01 ^b^	0.08 ± 0.00 ^d^	0.08 ± 0.00 ^d^
Σ SFA	11.41 ± 0.08 ^d^	6.65 ± 0.05 ^e^	18.15 ± 0.11 ^c^	23.96 ± 0.17 ^b^	43.96 ± 0.31 ^a^
Σ UFA	87.71 ± 0.62 ^b^	93.09 ± 0.66 ^a^	81.60 ± 0.15 ^c^	66.84 ± 0.47 ^d^	55.80 ± 0.39 ^e^
Σ MUFA	81.07 ± 0.57 ^a^	75.72 ± 0.54 ^b^	27.53 ± 0.06 ^e^	54.69 ± 0.39 ^c^	44.69 ± 0.32 ^d^
Σ PUFA	6.73 ± 0.05 ^e^	17.43 ± 0.12 ^b^	54.07 ± 0.08 ^a^	12.15 ± 0.09 ^c^	11.15 ± 0.08 ^d^
C18:2/C16:0	1.10	3.64	4.06	3.33	0.28

Values are means ± SD (*n* = 3). Values followed by different letters in the same row differed beyond 5% significance. SFA, saturated fatty acid; UFA, unsaturated fatty acid; MUFA, monounsaturated fatty acid; and PUFA, polyunsaturated fatty acids.

## Data Availability

The original contributions presented in the study are included in the article, further inquiries can be directed to the corresponding author.
